# Selenium-Enriched Soybean Peptides as Novel Organic Selenium Compound Supplements: Inhibition of Occupational Air Pollution Exposure-Induced Apoptosis in Lung Epithelial Cells

**DOI:** 10.3390/nu16010071

**Published:** 2023-12-25

**Authors:** Jian Zhang, Wenhui Li, He Li, Wanlu Liu, Lu Li, Xinqi Liu

**Affiliations:** 1 National Soybean Processing Industry Technology Innovation Center, Beijing Technology and Business University, Beijing 100048, China; tsnpzhj@163.com (J.Z.); lwhhuijiop@163.com (W.L.); liuwanlu19990115@163.com (W.L.); lil@btbu.edu.cn (L.L.); liuxinqi@btbu.edu.cn (X.L.); 2 Department of Nutrition and Health, China Agricultural University, Beijing 100193, China; 3 Beijing Engineering and Technology Research Center of Food Additives, Beijing Technology and Business University, Beijing 100048, China

**Keywords:** selenium-enriched soybean peptides, fine particulate matter, apoptosis, oxidative stress, inflammatory, protective effect

## Abstract

The occupational groups exposed to air pollutants, particularly PM2.5, are closely linked to the initiation and advancement of respiratory disorders. The aim of this study is to investigate the potential protective properties of selenium-enriched soybean peptides (Se-SPeps), a novel Se supplement, in mitigating apoptosis triggered by PM2.5 in A549 lung epithelial cells. The results indicate a concentration-dependent reduction in the viability of A549 cells caused by PM2.5, while Se-SPeps at concentrations of 62.5–500 µg/mL showed no significant effect. Additionally, the Se-SPeps reduced the production of ROS, proinflammatory cytokines, and apoptosis in response to PM2.5 exposure. The Se-SPeps suppressed the PM2.5-induced upregulation of Bax/Bcl-2 and caspase-3, while also restoring reductions in p-Akt in A549 cells. The antiapoptotic effects of Se-SPeps have been found to be more effective compared to SPeps, SeMet, and Na_2_SeO_3_ when evaluated at an equivalent protein or Se concentration. Our study results furnish evidence that supports the role of Se-SPeps in reducing the harmful effects of PM2.5, particularly in relation to its effect on apoptosis, oxidative stress, and inflammation.

## 1. Introduction

In recent years, the accelerated rate of industrialization and urbanization has given rise to a significant worldwide environmental issue: air pollution. Although attempts to regulate air pollution have resulted in some advancements in air quality, there are still specific occupations that necessitate humans working in environments characterized by significant air pollution [1]. In urban settings, the primary origins of PM2.5 are linked to air pollutants from transportation [2], with peak pollutant concentrations in transportation settings reaching up to three times higher than background levels [3]. Consequently, bus drivers, traffic police, sanitation workers, and other occupations are the main exposure groups of PM2.5 [4,5]. Various health problems, particularly the initiation and advancement of respiratory diseases, are closely associated with occupational exposure to PM2.5 [6]. PM2.5 first contacts the lung epithelial cells after entering the human body, inducing stimulation and destruction, increasing the release of ROS, and inducing oxidative damage in lung tissue [7]. In addition, it can further stimulate and induce the release of various proinflammatory cytokines, resulting in inflammation [8]. The damage and shedding of lung epithelial cells can aggravate the toxic effect of PM2.5, thereby leading to more severe inflammatory responses and lung injury. Prolonged exposure to low levels of PM2.5 has been shown to trigger oxidative stress and inflammation in healthy mice, resulting in apoptosis of bronchial and alveolar epithelial cells [9,10]. Zhang et al. demonstrated that PM2.5 prompted the apoptosis of A549 cells by upregulating the Bax/Bcl-2 ratio and the expression levels of caspase-3 [11]. Hence, the initiation of oxidative stress and inflammation by PM2.5 may result in the apoptosis of alveolar epithelial cells. However, utilizing barriers or filtration techniques to lessen particle inhalation is the primary strategy used presently to protect people from PM2.5 exposure [12]. Despite the aforementioned measures, inhaling PM2.5 is frequently unavoidable and has the potential to cause damage to lung cells. Hence, it is imperative to develop novel and effective strategies aimed at preventing or mitigating PM2.5-induced apoptosis and lung damage, as this is essential for safeguarding the health of individuals exposed to occupational hazards.

Natural foods to prevent PM2.5-induced lung damage have attracted a great deal of public interest. Selenium (Se) is considered a crucial microelement for promoting health, primarily owing to its antioxidant and anti-inflammatory properties [13]. Research has shown that administering Se can effectively safeguard rat lungs from acute injuries by activating GSH-Px, diminishing the inflammatory response, and reducing lipid peroxidation [14,15]. Se-containing compounds, which include sodium selenite and selenomethionine, along with selenoproteins and Se nanoparticles, possess the capacity to regulate defense systems against many viral infections, including COVID-19 [16]. Methylselenic acid can also control the cell cycle by influencing the PI3K/AKT/mTOR signaling pathway [17]. Hence, Se exhibits the potential to mitigate lung damage resulting from the inhalation of air pollutants. Se is incorporated into the molecular structure of selenoproteins within the human body, contributing to their biological functions [18]. Organic forms of Se are more effective in fulfilling dietary requirements compared to inorganic forms, due to improvements in bioavailability and low toxicity [19]. In recent years, researchers have become increasingly interested in extracting peptides from Se-enriched, plant-derived foods and determining their anti-inflammatory and antioxidant activities. Several investigations have demonstrated that peptides containing Se have displayed remarkable capabilities in terms of antioxidation and immunoregulation [20,21]. Research findings indicate that Se-enriched soybean peptides (Se-SPeps) possess the ability to mitigate oxidative damage induced by H_2_O_2_ through the enhancement of GSH-Px activity [22]. Fang et al. revealed that Se-enriched rice peptides exhibited high immunomodulatory activity. Soybean peptides (SPeps) have been proven to have anti-inflammatory and antioxidant properties [23]. The study results demonstrate that SPeps exhibited significant antioxidant properties in safeguarding HepG2 cells from oxidative stress induced by H_2_O_2_ [24]. In addition, it has been observed that SPeps exhibit a mitigating effect on the inflammatory response within the RAW264.7 cell provoked by LPS [25]. Based upon this literature, it is postulated that Se-SPep have the potential to mitigate the detrimental impacts of PM2.5 on A549 cell apoptosis by regulating oxidative stress and inflammatory responses.

Within this investigation, A549 cells were subjected to the intervention of Se-SPeps and exposed to PM2.5 in vitro. The analysis encompassed the examination of apoptosis, intracellular generation of ROS, secretion of proinflammatory factors, and expression of proteins related to apoptosis. This study aimed to explore the combined mechanism of Se-SPep intervention in countering oxidative stress, inflammatory response, and apoptosis triggered by PM2.5 in A549 cells.

## 2. Materials and Methods

### 2.1. Materials

Se-enriched soybeans were acquired from Enshi Se-Run Health Tech Development Co., Ltd. (Enshi, China). The PM2.5 standard reference material (SRM 2786) [26] was procured from the NIST (Gaithersburg, MD, USA). Seleno-DL-methionine (SeMet) and sodium selenite (Na_2_SeO_3_) were purchased from Sigma-Aldrich Co. (St. Louis, MO, USA). DMEM, FBS, penicillin, and streptomycin were acquired from Gibco (Grand Island, NE, USA). The ELISA kits for IL-1β, IL-6, and TNF-α were purchased from Multisciences (Hangzhou, China). The antibodies against Bax (AF1270), Bcl-2 (AF6285), caspase-3 (AF1213), Akt (AF1777), p-Akt (Ser473) (AA329), β-Actin (AF5003), and HRP-labeled goat anti-rabbit IgG (H + L) were obtained from Beyotime (Shanghai, China). The Hoechst 33342, ECL kit, CCK-8 kit, and ROS kit were supplied by Beyotime (Shanghai, China). The RIPA lysis buffer, Annexin V-FITC/PI apoptosis kit, and BCA protein assay kit were supplied by Solarbio (Beijing, China).

### 2.2. Preparation of the Se-SPeps

A previous study described a method for extracting Se-SPeps from Se-enriched soybeans [27]. In summary, the Se-enriched soybeans were subjected to grinding, defatting, and drying processes in order to obtain soybean kernel flour. Using soybean kernel flour as the raw material, Se-enriched soybean protein (Se-SPro) precipitate was obtained through alkali dissolution and acid precipitation. The Se-SPro precipitate was then redissolved, followed by dialysis using a 3500 Da membrane at 4 °C. The dialysate was freeze-dried to produce the Se-SPro. The Se-SPro underwent digestion in a 2:1:1 ratio with alkaline protease, neutral protease, and papain. Proteases, constituting 0.2% of the Se-SPro weight, were introduced and subjected to hydrolysis under optimal conditions at 50 °C for 4 h. Following this, the hydrolysate underwent centrifugation at 3500× *g* for 15 min. The resulting supernatant was freeze-dried to yield Se-SPeps. The procedure for SPep preparation mirrored the aforementioned process. The Se concentrations in Se-SPeps and SPeps were determined as 86.03 ± 4.28 mg/kg and 0.06 ± 0.03 mg/kg, respectively, utilizing hydride generation atomic fluorescence spectrometry (LCAFS6500, Beijing Haiguang Instrument Co., Ltd., Beijing, China) following the Chinese national standard GB 5009.93-2010 [28]. The protein concentrations of the Se-SPeps and SPeps were 85.10 ± 0.21% and 87.41 ± 0.04%, assessed using the Kjeldahl method. Essential amino acids constituted 36.88 ± 1.23% and 37.18 ± 1.17% of the total amino acids (Table S1) in Se-SPeps and SPeps, determined using an amino acid analyzer (Biochrom 30+, BioChrom Ltd., Cambridge, UK). Utilizing an ÄKTA pure system (AKTA pure 25, Cytiva, Marlborough, MA, USA) and following the procedure outlined in the Chinese national standard GB/T 22492-2008 [29], the Se-SPeps and SPeps demonstrated molecular weights (Figure S1) below 3000 Da, determined to be 86.42% and 88.46%, respectively.

### 2.3. Preparation of the PM2.5 Suspension

Briefly, the PM2.5 was obtained from NIST (SRM 2786) with an average particle diameter of 2.8 μm and was collected in 2005 in Prague, Czech Republic. The primary constituents consisted of trace elements, polycyclic aromatic hydrocarbons, and polybrominated diphenyl ether (Table S2). PM2.5 was suspended in DMEM medium without FBS, achieving a final concentration of 1 mg/mL. Subsequently, the suspension underwent sonication for 20 min before administration.

### 2.4. Cell Lines and Cell Culture

The A549 cells were purchased from the PCRC (Beijing, China). The A549 cells within 10 generations were thawed in 37 °C water bath and transferred to high glucose DMEM medium supplemented with 10% FBS and 1% penicillin/streptomycin. Following this, the cells underwent cultivation and revival within a cell incubator (37 °C, 5% CO_2_) for the ensuing cytotoxicity assessment.

### 2.5. Cytotoxicity of PM2.5 and Se-SPeps

The cell viability of A549 cells was assessed through the CCK-8 assay. Cells were planted in 96-well plates with a density of 5 × 10^3^ cells per well and incubated for 24 h. Next, the cells underwent a 24 h treatment with varying doses of PM2.5 (0, 25, 50, 100, 150, and 200 μg/mL) or Se-SPeps (0, 62.5, 125, 250, 500, 1000, 2000, and 4000 μg/mL), followed by 60 min at a 37 °C incubation with 10 μL CCK-8 solution. The microplate reader (Infinite 200 Pro Nanoquant, Tecan, Männedorf, Switzerland) measured absorbance at 450 nm. Expressing cell viability as a percentage, it compared the absorbance of treated cells to that of untreated cells. Concurrently, an inverted microscope (IX73, OLYMPUS, Tokyo, Japan) was employed to observe and capture images of cell morphology.

### 2.6. Toxicity Suppression by Se-SPeps

The protective effect of Se-SPeps was examined by plating A549 cells in 96-well plates with a density of 5 × 10^3^ cells per well and allowing them to culture for 24 h. The media was then replaced with DMEM media containing samples of 125, 250, and 500 μg/mL of Se-SPeps, SPeps (the same protein concentration as 250 μg/mL of Se-SPeps), SeMet (the same Se concentration as 250 μg/mL of Se-SPeps), and Na_2_SeO_3_ (the same Se concentration as 250 μg/mL of Se-SPeps) for 24 h. The Se concentration in the SeMet and Na_2_SeO_3_ groups was 0.022 μg/mL, which was equivalent to the Se concentration in the 250 μg/mL of Se-SPep group. After removing the samples, PM2.5 was added to the medium at a final concentration of 150 μg/mL and incubated for 24 h. The control group received an equal volume of DMEM, while the model group received an average amount of DMEM only containing PM2.5, and the cell viability test was performed using CKK-8.

### 2.7. Analysis of Cell Apoptosis

To assess the protective effect of Se-SPeps against PM2.5-induced cell apoptosis, annexin V-FITC/PI double staining was carried out. A549 cells were seeded at a density of 5 × 10^4^ cells per well in 24-well plates, then incubated without or with Se-SPeps, SPeps, SeMet, and Na_2_SeO_3_ for 24 h followed by PM2.5 treatment as previously described. After Se-SPep and PM2.5 treatments, the cells were suspended in 1 mL of binding buffer, accompanied by 5 μL of annexin V-FITC and 5 μL of PI, in darkness at 37 °C for 20 min. Flow cytometry was used to detect fluorescence immediately after. The treated cells were also resuspended with binding buffer supplemented with Hoechst 33342 for 30 min. A fluorescence microscope was used to monitor the apoptotic cells (IX73, OLYMPUS, Japan).

### 2.8. Detection of Intracellular ROS

The fluorogenic dye DCFH-DA was used to measure ROS production. The A549 cells were seeded at a density of 5 × 10^4^ cells per well in 24-well plates and then preincubated without or with Se-SPeps, SPeps, SeMet, and Na_2_SeO_3_ for 24 h followed by PM2.5 treatment as described previously. Following that, cells underwent a PBS solution wash and were subjected to a 30 min incubation with 10 μmol/L DCFA-DA at 37 °C. After incubation, excess DCFH-DA was eliminated by washing the cells with PBS, and 300 μL of PBS was introduced into each well. The flow cytometer (FACSAria III, BD, Franklin Lakes, NJ, USA) was employed to quantify fluorescence intensity, with the excitation wavelength set at 488 nm and the emission wavelength at 525 nm. Additionally, the cells were scrutinized using a fluorescence microscope (IX73, OLYMPUS, Japan).

### 2.9. Determination of the Cytokines

Supernatants devoid of cells were gathered following treatment with Se-SPeps and PM2.5. The concentrations of IL-1β, IL-6, and TNF-α in the supernatants were determined using ELISA kits.

### 2.10. Western Blot Analysis

A549 cells were seeded into 24-well plates and subsequently incubated for 24 h with Se-SPeps, SPeps, SeMet, and Na_2_SeO_3_ before being treated with PM2.5 treatment as previously described. After the treatments, PBS was used to wash A549 cells, followed by lysis on ice in RIPA buffer for 10 min, which included 1% PMSF and a cocktail of phosphatase inhibitors. Whole-cell lysates underwent centrifugation at 10,000× *g* for 5 min at 4 °C, leading to the collection of supernatants. The total protein concentration was gauged using the BCA protein quantitative kit. On a 10% polyacrylamide gel, equal amounts of protein (10 μg) were separated and transferred onto PVDF membranes. The membranes were blocked with 5% BSA at room temperature for 1 h. The PVDF membranes were incubated with primary antibodies against β-Actin, Akt, p-Akt, Bax, Bcl-2, and caspase-3 overnight at 4 °C. Following incubation, the membranes were washed three times with TBST and incubated with an HRP-labeled secondary antibody for 1 h at room temperature. After being rewashed with TBST, the bands were developed by an ECL kit. The gray value of the protein bands was analyzed using ImageJ vision 1.8.0 software. The relative expression of the target protein was calculated using the following formula: (the target protein/gray value of β-Actin).

### 2.11. Statistical Analysis

The mean ± SD represented the data. Statistical analysis was performed utilizing ANOVA followed by Tukey’s test with SPSS 23 software. Graphs were generated using Graph Pad Prism 9.2.0 software.

## 3. Results and Discussion

### 3.1. Effect of PM2.5 on the Viability of A549 Cells

**Figure 1:**
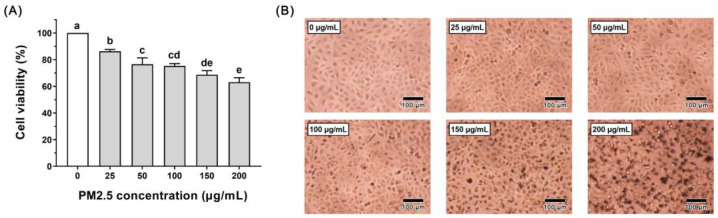
Effect of different concentrations (0, 25, 50, 100, 150, and 200 μg/mL) of PM2.5 pretreatment on A549 cells for 24 h. (**A**) Viability of the A549 cells. (**B**) Cell morphological changes of the A549 cells. Scale bar = 100 μm. Data are shown as the mean ± SD, *n* = 8/group. Statistical analysis was performed using ANOVA followed by Tukey’s post hoc test. Different letters over bars indicate statistically significant differences (*p* < 0.05).

We investigated the detrimental impacts of PM2.5 on A549 cells. The A549 cells were exposed to different concentrations of PM2.5 (0, 25, 50, 100, 150, and 200 μg/mL) for 24 h. The CCK-8 assay demonstrated that the incubation of A549 cells with 25–200 µg/mL PM2.5 for 24 h reduced their viability in a concentration-dependent manner (Figure 1A), with a significant difference between each dose group and the 0 μg/mL group (*p* < 0.05). At concentrations exceeding 50 μg/mL, the cell viability dropped below 80%, indicating that PM2.5 had a strong toxic effect. At the highest concentration of 200 μg/mL, the cell viability was only 63.05%. The microscopic observation results are shown in Figure 1B. The cells in the 0 μg/mL group have a complete spindle shape and uniform distribution. At concentrations surpassing 150 μg/mL, A549 cells adhered to the PM2.5 particles, and the cell membrane boundary became indiscernible, resulting in evident damage and death. Based on these results, we selected a concentration of PM2.5 of 150 μg/mL, which led to a cell viability of 68.68%. We chose this concentration with the rationale of utilizing a midpoint effective dose, inducing damage to the cell population without triggering a complete collapse.

### 3.2. Effect of Se-SPeps on the Viability of A549 Cells

**Figure 2:**
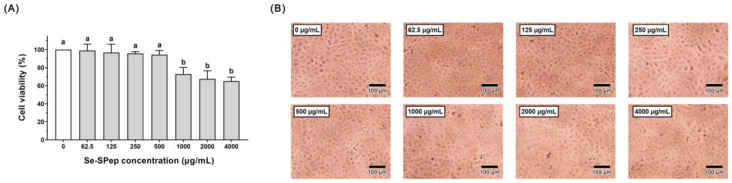
Effect of different concentrations (0, 62.5, 125, 250, 500, 1000, 2000, and 4000 μg/mL) of Se-SPep pretreatment on A549 cells for 24 h. (**A**) Viability of the A549 cells. (**B**) Cell morphological changes of the A549 cells. Scale bar = 100 μm. Data are shown as the mean ± SD, *n* = 8/group. Statistical analysis was performed using ANOVA followed by Tukey’s post hoc test. Different letters over bars indicate statistically significant differences (*p* < 0.05).

To assess the Se-SPep’s potential as a therapeutic intervention against PM2.5-induced toxicity in A549 cells, it was essential to determine a dose range that would not adversely affect the cells. Although Se and peptides exhibit documented benefits to human health, certain reports have indicated potential adverse effects when Se is present in high concentrations [30]. Hence, A549 cells were exposed to varying concentrations of Se-SPeps (0, 62.5, 125, 250, 500, 1000, 2000, and 4000 μg/mL). Subsequently, the cell viability was quantified to determine the optimal concentration for our assays. Illustrated in Figure 2A, the CCK-8 assay revealed that exposure to Se-SPep concentrations ranging from 62.5 to 500 µg/mL for 24 h did not induce a noteworthy impact on the viability of A549 cells. The viability of A549 cells was 99.04%, 96.83%, 95.76%, and 94.37% when the Se-SPep concentration was below 500 μg/mL compared to the 0 μg/mL group, respectively. When the concentration of Se-SPeps was 1000, 2000, and 4000 μg/mL, the cell viability decreased to 72.93%, 67.57%, and 65.18%, respectively. The results demonstrated that Se-SPeps are cytotoxic at high concentrations (more than 1000 μg/mL). Furthermore, to determine the optimal Se-SPep concentration, the cell morphology was examined. Illustrated in Figure 2B, cells in the low-concentration Se-SPep treatment group (less than 500 μg/mL) were in a normal state, and no significant decrease in the cell number was observed compared to the 0 μg/mL group. Treatment with high doses of Se-SPeps (over 1000 μg/mL) resulted in cell damage and decreased cell numbers. Based on the cell viability assessment and cell state observation, 125, 250, and 500 μg/mL were selected as the treatment concentrations of Se-SPeps for subsequent experiments.

### 3.3. Effect of Se-SPeps on the Viability of A549 Cells Exposed to PM2.5

**Figure 3:**
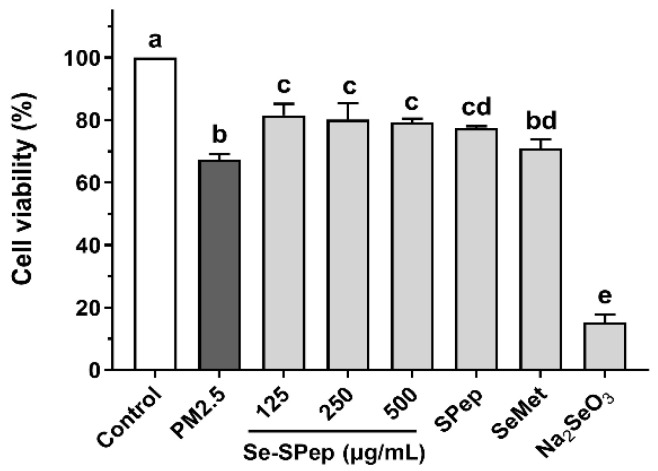
Effect of Se-SPep pretreatment on the cell viability in A549 cells exposed to PM2.5. The Se concentration in the SeMet and Na_2_SeO_3_ groups is 0.022 μg/mL, which is equivalent to the Se concentration in the 250 μg/mL Se-SPep group. Data are shown as the mean ± SD, *n* = 8/group. Statistical analysis was performed using ANOVA followed by Tukey’s post hoc test. Different letters over bars indicate statistically significant differences (*p* < 0.05).

After the Se-SPep intervention, A549 cells encountered exposure to PM2.5 to explore the protective impact of Se-SPeps on their viability following injury induced by PM2.5. As shown in Figure 3, the 150 µg/mL of PM2.5 significantly decreased the viability of the DMEM-pretreated cells compared to control cells (*p* < 0.05). However, the cell viabilities of Se-SPep- and SPep-pretreated cells, after PM2.5 exposure, were markedly higher than those of DMEM-pretreated cells (*p* < 0.05). The SeMet group exhibited a higher cell viability than the PM2.5 group, yet the disparity lacked statistical significance (*p* > 0.05). Contrarily, the Na_2_SeO_3_ group witnessed a significant decrease in cell viability (*p* < 0.05), reaching 15.22%, indicating that Na_2_SeO_3_ pretreatment had a significant toxic effect on A549 cells. The findings underscore the Se-SPep’s protective influence on viability against the PM2.5-induced injury in A549 cells, with the organic Se form outperforming the inorganic Se form.

### 3.4. Effect of Se-SPeps on Cell Apoptosis of A549 Cells Exposed to PM2.5

**Figure 4:**
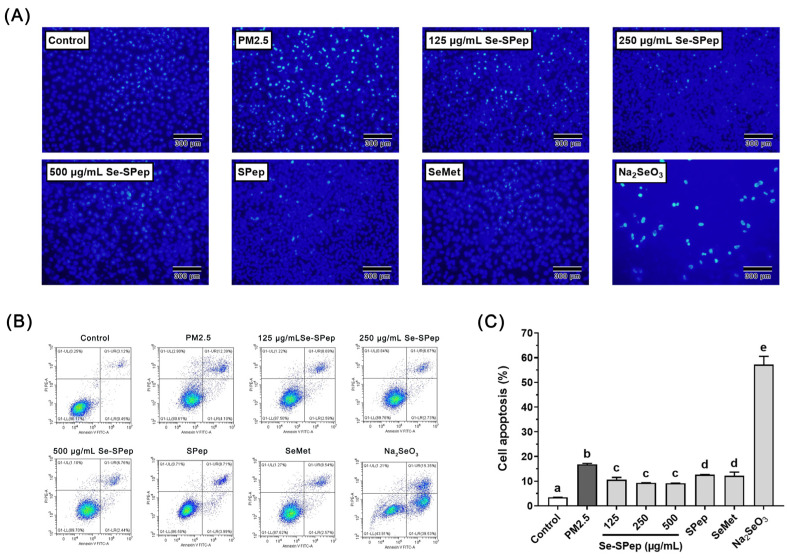
Effect of Se-SPep pretreatment on cell apoptosis in A549 cells exposed to PM2.5. (**A**) Representative photomicrographs of A549 cells stained with Hoechst 33342 fluorescent dye. Scale bar = 300 μm. (**B**) Flow cytometry analysis of cell apoptosis induced by PM2.5. (**C**) The apoptosis ratio induced by PM2.5 after intervention with Se-SPeps. The Se concentration in the SeMet and Na_2_SeO_3_ groups is 0.022 μg/mL, which is equivalent to the Se concentration in the 250 μg/mL Se-SPep group. Data are shown as the mean ± SD, *n* = 3/group. Statistical analysis was performed using ANOVA followed by Tukey’s post hoc test. Different letters over bars indicate statistically significant differences (*p* < 0.05).

A distinctive feature of PM2.5 toxicity is its ability to directly affect cells, leading to apoptosis [31]. To assess the protective effect of Se-SPeps on PM2.5-induced apoptosis in A549 cells, the Hoechst 33342 and annexin V-FITC assays were employed. Illustrated in Figure 4A, the PM2.5 group exhibited a pronounced increase in potent blue fluorescence compared to the control group. Simultaneously, a noteworthy reduction in blue fluorescence was observed after Se-SPep, SPep, and SeMet interventions compared to the PM2.5 group. A few viable cells were stained in the Na_2_SeO_3_ group, and a more intense blue fluorescence was observed. As shown in Figure 4B,C, the flow cytometry analysis revealed a notable rise in the apoptotic percentage in the PM2.5 group (*p* < 0.05), reaching 16.81% compared to the control group. This implies a substantial induction of cell apoptosis by PM2.5. In contrast, Se-SPep, SPep, and SeMet treatments significantly decreased the apoptotic cell percentages to 10.64%, 9.39%, 9.21%, 12.62%, and 12.19%, respectively. The Se-SPep group had significantly lower cell apoptosis than the SPep and SeMet groups (*p* < 0.05). Na_2_SeO_3_ intervention induced a noteworthy elevation in the apoptotic rate of A549 cells, reaching 57.22%. The results showed that Se-SPeps could prevent the apoptosis of A549 cells induced by PM2.5. The antiapoptotic ability of Se-SPeps was greater than that of SPeps and SeMet.

### 3.5. Effect of Se-SPeps on ROS Generation of A549 Cells Exposed to PM2.5

Research findings have offered substantiation that prolonged exposure to PM2.5 can induce respiratory system damage [32,33]. Oxidative stress, a crucial mechanism, is implicated in the induction of damage to the respiratory system, representing a significant factor in the negative impacts of PM2.5 air pollution on respiratory health [34]. Cells produce a large amount of ROS when exposed to PM2.5, causing oxidative damage to tissues and cells, triggering cellular inflammatory reaction and apoptosis [35,36]. Lao et al. reported that oxidative stress occurred in A549 cells after exposure to airborne PM2.5 elements [37]. Further investigations have demonstrated a significant increase in ROS levels in bronchial epithelial cells following exposure to airborne PM2.5 components [38]. In addition, a decreased cell viability caused by PM2.5 exposure was correlated with ROS overproduction.

**Figure 5:**
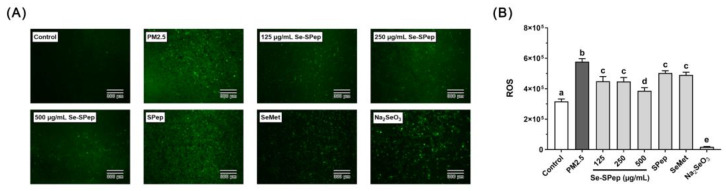
Effect of Se-SPep pretreatment on ROS generation in A549 cells exposed to PM2.5. (**A**) Representative photomicrographs of A549 cells stained with DCFH-DA fluorescent dye. Scale bar = 300 μm. (**B**) Relative fluorescence intensity of intracellular ROS of A549 cells. The Se concentration in the SeMet and Na_2_SeO_3_ groups is 0.022 μg/mL, which is equivalent to the Se concentration in the 250 μg/mL Se-SPep group. Data are shown as the mean ± SD, *n* = 3/group. Statistical analysis was performed using ANOVA followed by Tukey’s post hoc test. Different letters over bars indicate statistically significant differences (*p* < 0.05).

As shown in Figure 5A, DCFH-DA detection reveals that ROS green fluorescence in the control group is virtually invisible, whereas the green fluorescence in the PM2.5 group surpasses that of the control group significantly. Meanwhile, Figure 5B shows that ROS fluorescence intensity in the PM2.5 group is 1.8 times higher than that in the control group, indicating that PM2.5 induces a strong intracellular oxidative stress response in cells. The intracellular green fluorescence and DCF fluorescence intensities were significantly lower after Se-SPep, SPep, and SeMet intervention compared to the PM2.5 group (*p* < 0.05). The ROS level in the 500 µg/mL of Se-SPep group was markedly lower than that in the SPep group (*p* < 0.05). However, due to apoptosis, both the cell count and fluorescence intensity in the Na_2_SeO_3_ group were significantly decreased (*p* < 0.05). These results suggest that Se-SPeps, SPeps, and SeMet inhibit the generation of intracellular ROS, protecting A549 cells from oxidative stress generated by PM2.5 exposure and preventing cell apoptosis. Simultaneously, Se-SPeps have a more significant inhibitory effect on PM2.5-induced cellular oxidative stress. This effect was amplified when Se was combined with SPeps, indicating that Se-SPeps can suppress ROS generation, which is beneficial for cell viability.

### 3.6. Effect of Se-SPeps on Proinflammatory Cytokines Release of A549 Cells Exposed to PM2.5

**Figure 6:**
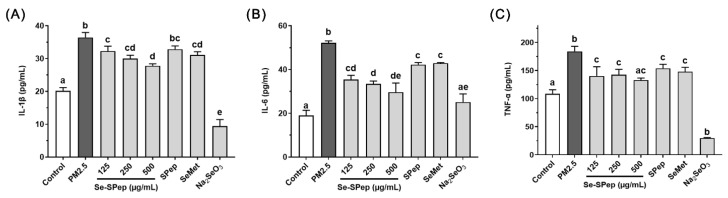
Effect of Se-SPep pretreatment on proinflammatory cytokines secretion in A549 cells exposed to PM2.5. (**A**) IL-1β secretion. (**B**) IL-6 secretion. (**C**) TNF-α secretion. The Se concentration in the SeMet and Na_2_SeO_3_ groups is 0.022 μg/mL, which is equivalent to the Se concentration in the 250 μg/mL Se-SPep group. Data are shown as the mean ± SD, *n* = 3/group. Statistical analysis was performed using ANOVA followed by Tukey’s post hoc test. Different letters over bars indicate statistically significant differences (*p* < 0.05).

Respiratory diseases exhibit a close association with inflammation. PM2.5 can stimulate lung immune cells to initiate an acute inflammatory response, stimulate normal cells to secrete numerous proinflammatory cytokines, modulate the inflammatory response, and induce inflammatory damage [39,40]. The inflammatory response induced by PM2.5 has been assessed by measuring the levels of expression of various proinflammatory cytokines. Figure 6 illustrates the ability of Se-SPeps to alleviate the release of IL-1β, IL-6, and TNF-α from PM2.5-induced A549 cells. The levels of proinflammatory cytokines IL-1β, IL-6, and TNF-α in the PM2.5 group were significantly increased in comparison to the control group (*p* < 0.05). The findings demonstrate that PM2.5 has the ability to induce multiple proinflammatory cytokines and inflammatory responses in A549 cells, proving that exposure to PM2.5 is immunotoxic to A549 cells. Meanwhile, after Se-SPep and SeMet intervention, the intracellular levels of IL-1β, IL-6, and TNF-α exhibited a significant decrease in comparison to the PM2.5 group (*p* < 0.05). The intervention with SPeps resulted in notably lower levels of IL-6 and TNF-α compared to the PM2.5 group (*p* < 0.05). In particular, the levels of IL-6 in the Se-SPep group exhibited a significant reduction compared to the SPep group (*p* < 0.05), while the levels of IL-1β and TNF-α in the Se-SPep group were lower than those in the SPep group (*p* > 0.05). Concentrations of proinflammatory cytokines in the Na_2_SeO_3_ group were notably lower due to severe apoptosis.

PM2.5 has the potential to induce and exacerbate inflammatory responses in cells, exerting toxic effects on cellular function [41,42]. Several studies have indicated that PM2.5 has the capacity to elevate the secretion of proinflammatory cytokines [43,44], indicating that inflammation is a crucial mechanism of cellular damage in the respiratory system. Xue et al. demonstrated that PM2.5 could significantly promote the release of proinflammatory factors in cells and lead to cell damage [45]. In alignment with the outcomes of this investigation, PM2.5 has the capacity to induce an inflammatory response in A549 cells, elevating the secretion of proinflammatory cytokines such as IL-1β, IL-6, and TNF-α. The results show that after the intervention of Se-SPeps, SPeps, and SeMet, the proinflammatory cytokines decrease significantly compared with the PM2.5 group, proving that the intervention of Se-SPeps, SPeps, and SeMet could inhibit the PM2.5-induced inflammatory damage of cells. In addition, Se-SPeps exhibited a more robust capability to inhibit the production of proinflammatory cytokines compared to SPeps.

### 3.7. Effect of Se-SPeps on the Mitochondrial Apoptotic Pathway of A549 Cells Exposed to PM2.5

**Figure 7:**
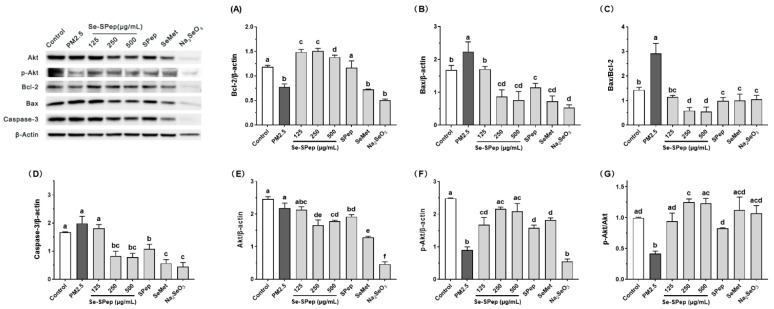
Effect of Se-SPep pretreatment on the mitochondrial apoptotic pathway in A549 cells exposed to PM2.5. The expression of Bcl-2, Bax, caspase-3, Akt, and p-AKT in PM2.5-exposed A549 cells was analyzed using Western blotting. β-Actin was used as the internal control. (**A**) Protein expression of Bcl-2. (**B**) Protein expression of Bax. (**C**) Protein expression of Bax relative to Bcl-2. (**D**) Protein expression of caspase-3. (**E**) Protein expression of Akt. (**F**) Protein expression of p-Akt. (**G**) Protein expression of p-AKT relative to AKT. The Se concentration in the SeMet and Na_2_SeO_3_ groups is 0.022 μg/mL, which is equivalent to the Se concentration in the 250 μg/mL Se-SPep group. Data are shown as the mean ± SD, *n* = 3/group. Statistical analysis was performed using ANOVA followed by Tukey’s post hoc test. Different letters over bars indicate statistically significant differences (*p* < 0.05).

According to our studies, when A549 cells are stimulated by PM2.5, a significant quantity of ROS is created, an inflammatory response is initiated, and proinflammatory cytokines are released, ultimately leading to apoptosis. The modulation of various proteins in the mitochondrial apoptosis pathway governs the intricate process of cellular apoptosis. Currently, the Bcl-2 family is the most studied of the apoptosis pathway-related proteins and is mainly classified as apoptosis-promoting and antiapoptotic [46]. The Bcl-2 family plays a pivotal role in the endogenous apoptosis pathway [47]. It was found that the ratio of Bax to Bcl-2 was the main factor determining the inhibitory effect on apoptosis [48]. An elevation in the Bax to Bcl-2 ratio modifies the potential of the mitochondrial membrane, stimulates the cytosolic release of cytochrome C, and triggers caspase-3 activation, ultimately leading to apoptosis [49]. Research has indicated that the oxidative stress induced by PM2.5 can disturb the antioxidant system and facilitate apoptosis through mitochondria-dependent mechanisms [50]. Therefore, regulating the expression of antiapoptotic and proapoptotic proteins within the Bcl-2 family is crucial for apoptosis control. The protein expression of antiapoptotic protein Bcl-2 and proapoptotic protein Bax in the intervention of Se-SPeps to prevent cell apoptosis was verified. Displayed in Figure 7A–C, the exposure to PM2.5 notably reduced the expression of the antiapoptotic protein Bcl-2 (*p* < 0.05), increased the expression of the proapoptotic protein Bax (*p* < 0.05), and elevated the Bax/Bcl-2 ratio significantly (*p* < 0.05). Zhang et al. proved that PM2.5-induced apoptosis could lead to an elevation in the Bax/Bcl-2 ratio [11]. We also found that PM2.5 could induce an increase in Bax while Bcl-2 levels experienced a significant decrease, highlighting Bax/Bcl-2 as a pathway for PM2.5-induced apoptosis in A549 cells. In the cells treated with Se-SPeps and SPeps, the Bax protein level was significantly decreased (*p* < 0.05) compared to the PM2.5 group, while the Bcl-2 protein level was significantly increased (*p* < 0.05), resulting in a decreased Bax/Bcl-2 ratio (*p* < 0.05). Meanwhile, the Se-SPep group exhibited significantly higher levels of the antiapoptotic protein Bcl-2 compared to the SPep and SeMet groups (*p* < 0.05), along with a lower Bax/Bcl-2 ratio than the SPep and SeMet groups (*p* < 0.05). These findings suggest that Se-SPep and SPep intervention can prevent PM2.5-induced apoptosis in A549 cells by regulating Bax/Bcl-2 protein expression. Secondly, the Se-SPeps have a more solid ability than the SPeps and SeMet to control the expression of Bax/Bcl-2 protein.

Caspase-3, identified as the “death executor protease”, acts as a pivotal player in apoptosis execution, amplifying signals from the caspase promoter and instigating apoptosis [51]. The activation of caspase-3 is considered to be a central link to apoptosis [52]. Illustrated in Figure 7D, the activation level of caspase-3 surged in cells exposed to PM2.5, signifying that PM2.5 exposure heightened apoptosis. However, the expression of caspase-3 significantly decreased in cells treated with Se-SPeps, SPeps, and SeMet compared to the PM2.5 group (*p* < 0.05). These findings imply that Se-SPeps, SPeps, and SeMet can protect A549 cells from apoptosis caused by an increased caspase-3 expression induced by PM2.5 exposure.

Research has indicated that the increased phosphorylation of Akt is related to the inhibition of apoptosis [53]. Akt can phosphorylate target proteins through multiple downstream pathways to play an antiapoptotic role [54]. The activation of this signaling pathway necessitates the initiation of S473 phosphorylation in a hydrophobic sequence [55]. Bcl-2 can depolymerize with phosphorylated BAD when Akt is activated, and free Bcl-2 can play an antiapoptotic role [56]. Figure 7E–G indicates that exposure to PM2.5 significantly decreased the phosphorylation level of Akt (*p* < 0.05). Intervention with Se-SPeps, SPeps, and SeMet markedly elevated the phosphorylation level of Akt (*p* < 0.05) in comparison to the PM2.5 exposure group. These findings suggest that Se-SPeps, SPeps, and SeMet may promote the antiapoptotic effect of A549 cells by increasing Akt phosphorylation. Meanwhile, the Se-SPeps had a more significant effect on Akt phosphorylation than the SPeps (*p* < 0.05), indicating a stronger antiapoptotic effect. The study showed that PM2.5 exposure inhibited Akt phosphorylation in A549 cells, which is consistent with the present findings [57]. These findings suggest that the decreased viability and apoptosis of A549 cells induced by PM2.5 are related to the activation of the mitochondrial apoptosis pathway triggered by oxidative stress and inflammatory response.

## 4. Conclusions

In this study, we investigated the protective effects of Se-SPeps on PM2.5-induced apoptosis via regulating oxidative stress and inflammatory response. The results demonstrated that Se-SPep intervention could prevent PM2.5-induced apoptosis and maintain regular cell morphology. Simultaneously, Se-SPep intervention can protect cells from the oxidative stress response caused by PM2.5 exposure by inhibiting the generation of intracellular ROS. Regarding the inflammatory response, Se-SPep intervention inhibited the overproduction of the proinflammatory cytokines IL-1β, IL-6, and TNF-α, thereby inhibiting the inflammatory damage caused by PM2.5 and reducing cell apoptosis. The intervention of Se-SPeps can play an antiapoptotic role by promoting the phosphorylation of Akt and the depolymerization of the Bcl-2 protein. Se-SPeps can also inhibit the proapoptotic effect of Bax by inhibiting the expression of the Bax protein. In addition, Se-SPep intervention inhibits the expression of caspase-3, thereby inhibiting its proapoptotic effects. This study also showed that Se-SPeps were more effective than SPeps in regulating the expression of Akt/Bcl-2/Bax pathway proteins to exert antiapoptotic effects. Finally, this study proved that nutritional intervention in the form of Se-SPeps and SeMet was safer compared to the primarily inorganic Se form of Na_2_SeO_3_. Our study demonstrates the effectiveness of Se-SPeps, a novel organic Se oral supplement, in providing protective effects against occupational air pollution-induced apoptosis of lung epithelial cells.

## Data Availability

The data presented in this study are available on request from the corresponding author.

## Supplementary Materials

The following supporting information can be downloaded at: https://www.mdpi.com/article/10.3390/nu16010071/s1, Figure S1: Sephadex G-25 chromatograms of the standard molecular weight samples of Se-SPep (A) and SPep (B); Table S1: The amino acid compositions in Se-SPep and SPep; Table S2: Mean concentration (μg/g) of main compositions detected in PM2.5 sample.
