# Supplementary material for: Selenium-Enriched Soybean Peptides as Novel Organic Selenium Compound Supplements: Inhibition of Occupational Air Pollution Exposure-Induced Apoptosis in Lung Epithelial Cells
Source: Nutrients. 2023 Dec 25;16(1):71. doi: 10.3390/nu16010071 (PMC10780830; doi:10.3390/nu16010071)
Supplement: Supplementary file 1 [file nutrients-16-00071-s001.zip › nutrients-2691270-supplementary.pdf]

Online Supplemental Materials

Selenium-Enriched Soybean Peptides as Novel Organic Selenium Compound Supplements: Inhibition of Occupational Air Pollution Exposure-Induced Apoptosis in Lung Epithelial Cells

Jian Zhang, Wenhui Li, He Li *, Wanlu Liu, Lu Li and Xinqi Liu

Table S1. The amino acid compositions in Se-SPep and SPep.

Amino Acid	Se-SPep (%)	SPep (%)
Aspartic acid[a]	11.36 ± 0.18	10.52 ± 1.29
Serine[a]	4.28 ± 0.08	3.72 ± 0.04
Glutamic acid[a]	23.87 ± 0.26	19.65 ± 0.88
Proline[a]	4.77 ± 0.13	8.08 ± 0.19
Glycine[a]	3.96 ± 0.07	3.70 ± 0.12
Alanine[a]	3.70 ± 0.08	3.72 ± 0.15
Cysteine[a]	0.25 ± 0.06	0.69 ± 0.03
Tyrosine[a]	3.84 ± 0.37	3.61 ± 0.08
Arginine[a]	7.10 ± 0.23	9.12 ± 0.44
Threonine[b]	3.23 ± 0.16	2.04 ± 0.36
Valine[b]	5.69 ± 0.22	5.47 ± 0.25
Methionine[b]	1.39 ± 0.18	0.91 ± 0.01
Isoleucine[b]	4.55 ± 0.17	4.38 ± 0.26
Leucine[b]	6.99 ± 0.07	8.42 ± 0.38
Phenylalanine[b]	5.13 ± 0.09	5.53 ± 0.22
Histidine[b]	2.88 ± 0.15	2.76 ± 0.16
Lysine[b]	7.02 ± 0.19	7.67 ± 0.44

Values are expressed as means ± SD (n=3). [a] Non-essential amino acids, [b] Essential amino acids.

Table S2. Mean concentration (μg/g) of main compositions detected in PM2.5 sample

	Component	Concentration	Component	Concentration
Trace Elements	Total Mercury	5.32	Copper	847
	Aluminum	33480	Lead	286
	Cadmium	4.34	Manganese	780
	Chromium	462.2	Vanadium	85.5
PAH	Fluorene	0.195	Benzo[a]pyrene	3.7
	Fluoranthene	10.28	Perylene	0.769
	Pyrene	8.01	Benzo[ghi]perylene	5.6
	Benzo[ghi]fluoranthene	3.158	Indeno[1,2,3-cd]pyrene	4.87
	Benzo[c]phenanthrene	1.597	Dibenz[a,c]anthracene	0.509
	Benz[a]anthracene	4.82	Dibenz[a,j]anthracene	0.61
	Chrysene	6.82	Dibenz[a,h]anthracene	0.717
	Triphenylene	1.794	Benzo[b]chrysene	0.662
	Benzo[b]fluoranthene	7.51	Picene	1.242
	Benzo[j]fluoranthene	4.37	Coronene	2.156
	Benzo[k]fluoranthene	3.48	Dibenzo[b,k]fluoranthene	1.013
	Benzo[a]fluoranthene	0.898	Dibenzo[a,e]pyrene	0.812
	Benzo[e]pyrene	4.77		
PBDE	PBDE 99	0.0076	PBDE 209	0.243

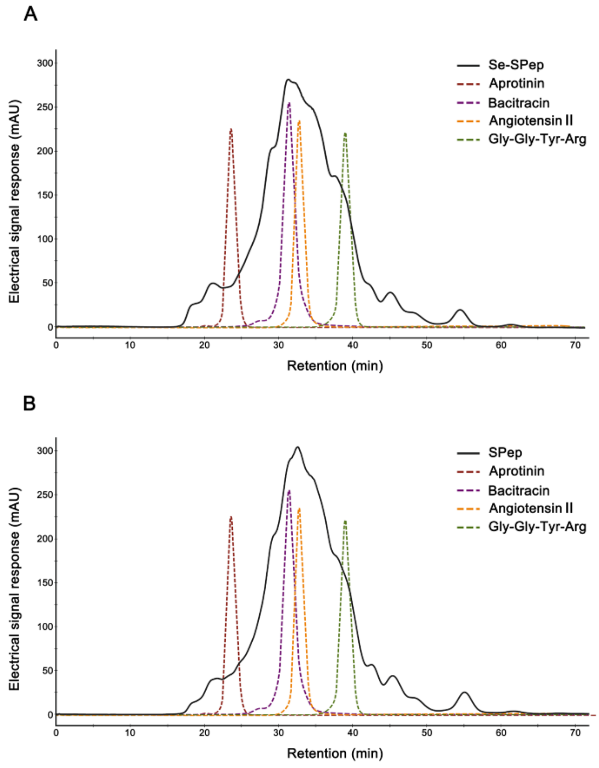

Figure S1. Sephadex G-25 chromatograms of the standard molecular weight samples of Se-SPep (A) and SPep (B). The standard molecular weight samples: Aprotinin-6511 Da, Bacitracin-1422 Da, Angiotensin II-1046 Da, and Gly-Gly-Tyr-Arg-451 Da.
